# 3-Amino-1*H*-pyrazol-2-ium tri­fluoro­acetate

**DOI:** 10.1107/S1600536813022204

**Published:** 2013-08-14

**Authors:** T. S. Yamuna, Jerry P. Jasinski, Derek R. Scadova, H. S. Yathirajan, Manpreet Kaur

**Affiliations:** a Department of Studies in Chemistry, University of Mysore, Manasagangotri, Mysore 570 006, India; b Department of Chemistry, Keene State College, 229 Main Street, Keene, NH 03435-2001, USA

## Abstract

The asymmetric unit of the title salt, C_3_H_6_N_3_^+^·C_2_F_3_O_2_^−^, contains two independent 3-amino­pyrazolium cations and two independent tri­fluoro­acetate anions. The F atoms of both anions were refined as disordered over two sets of sites, with common occupancy ratios of 0.639 (12):0.361 (12). In the crystal, the cations and anions are linked *via* N—H⋯O hydrogen bonds, forming chains along [100] and [010].

## Related literature

For biological properties of pyrazole derivatives, see: Hall *et al.* (2008 ▶); Isloor *et al.* (2009 ▶); Patel *et al.* (2010 ▶); Samshuddin *et al.* (2010 ▶). For the chemistry of amino­pyrazoles, see: Giuseppe *et al.* (1991 ▶). For the medicinal activity of pyrazoles, see: Vinogradov *et al.* (1994 ▶). For related structures, see: Dobson & Gerkin (1998 ▶); Foces-Foces *et al.* (1996 ▶); Hemamalini & Fun (2010 ▶); Thanigaimani *et al.* (2012 ▶). For hydrogen-bond graph-set motifs, see: Bernstein *et al.* (1995 ▶). For standard bond lengths, see: Allen *et al.* (1987 ▶).
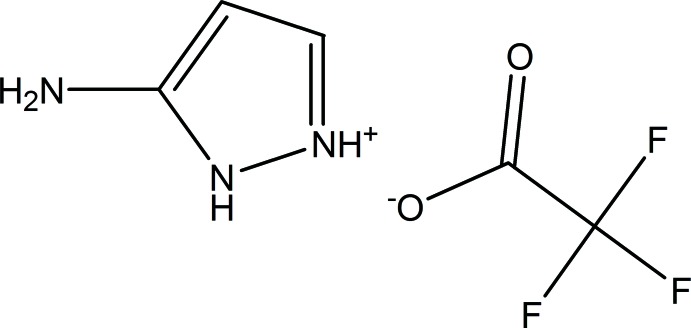


## Experimental

### 

#### Crystal data

C_3_H_6_N_3_^+^·C_2_F_3_O_2_^−^
*M* *_r_* = 197.13
Monoclinic, 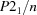

*a* = 10.9292 (8) Å
*b* = 10.9332 (6) Å
*c* = 13.7002 (13) Å
β = 107.939 (9)°
*V* = 1557.5 (2) Å^3^
*Z* = 8
Cu *K*α radiation
μ = 1.58 mm^−1^
*T* = 173 K
0.16 × 0.14 × 0.06 mm

#### Data collection

Agilent Xcalibur (Eos, Gemini) diffractometer
Absorption correction: multi-scan (*CrysAlis PRO* and *CrysAlis RED*; Agilent, 2012 ▶) *T*_min_ = 0.662, *T*_max_ = 1.000
9227 measured reflections
3031 independent reflections
2343 reflections with *I* > 2σ(*I*)
*R*_int_ = 0.030

#### Refinement

*R*[*F* ^2^ > 2σ(*F* ^2^)] = 0.048
*wR*(*F* ^2^) = 0.136
*S* = 1.05
3031 reflections
338 parameters
All H-atom parameters refined
Δρ_max_ = 0.24 e Å^−3^
Δρ_min_ = −0.23 e Å^−3^

### 

Data collection: *CrysAlis PRO* (Agilent, 2012 ▶); cell refinement: *CrysAlis PRO*; data reduction: *CrysAlis RED* (Agilent, 2012 ▶); program(s) used to solve structure: *SUPERFLIP* (Palatinus & Chapuis, 2007 ▶); program(s) used to refine structure: *SHELXL2012* (Sheldrick, 2008 ▶); molecular graphics: *OLEX2* (Dolomanov *et al.*, 2009 ▶); software used to prepare material for publication: *OLEX2*.

## Supplementary Material

Crystal structure: contains datablock(s) I. DOI: 10.1107/S1600536813022204/lh5637sup1.cif

Structure factors: contains datablock(s) I. DOI: 10.1107/S1600536813022204/lh5637Isup2.hkl

Supplementary material file. DOI: 10.1107/S1600536813022204/lh5637Isup3.cml

Additional supplementary materials:  crystallographic information; 3D view; checkCIF report

## Figures and Tables

**Table 1:** Hydrogen-bond geometry (Å, °)

*D*—H⋯*A*	*D*—H	H⋯*A*	*D*⋯*A*	*D*—H⋯*A*
N1*A*—H1*AA*⋯O1*A* ^i^	0.85 (3)	2.28 (3)	2.936 (3)	134 (2)
N1*A*—H1*AB*⋯O2*A* ^ii^	0.91 (3)	1.99 (3)	2.884 (3)	169 (3)
N2*A*—H2*AA*⋯O1*A* ^ii^	0.94 (3)	1.85 (3)	2.778 (2)	171 (3)
N3*A*—H3*AA*⋯O2*A*	0.93 (3)	1.78 (3)	2.705 (2)	172 (3)
N1*B*—H1*BA*⋯O2*B* ^iii^	0.84 (3)	2.18 (3)	2.962 (2)	153 (2)
N1*B*—H1*BB*⋯O2*B* ^iv^	0.90 (3)	2.03 (3)	2.929 (3)	173 (2)
N2*B*—H2*BA*⋯O1*B* ^iv^	0.95 (3)	1.81 (3)	2.756 (2)	174 (2)
N3*B*—H3*BA*⋯O1*B* ^v^	0.91 (3)	1.82 (3)	2.728 (2)	171 (2)

Symmetry codes: (i) 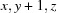; (ii) 
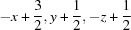
; (iii) 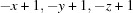; (iv) 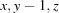; (v) 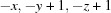.

**Fig. 1.**
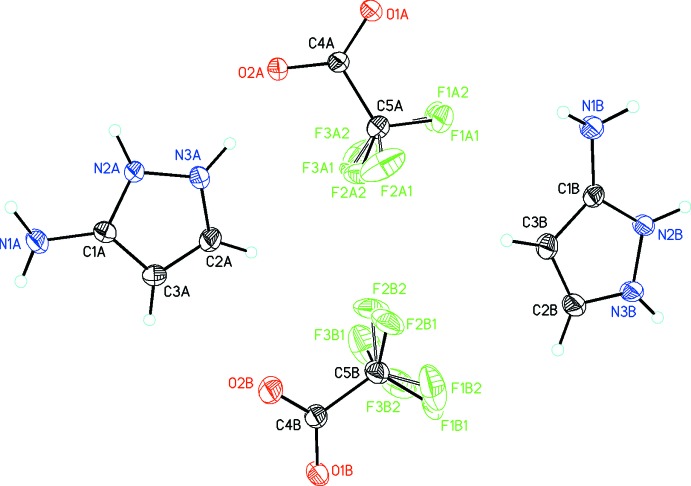
The asymmetric unit of the title compound. Displacement ellipsoids are drawn at the 30% probability level. All disorder components are shown.

**Fig. 2.**
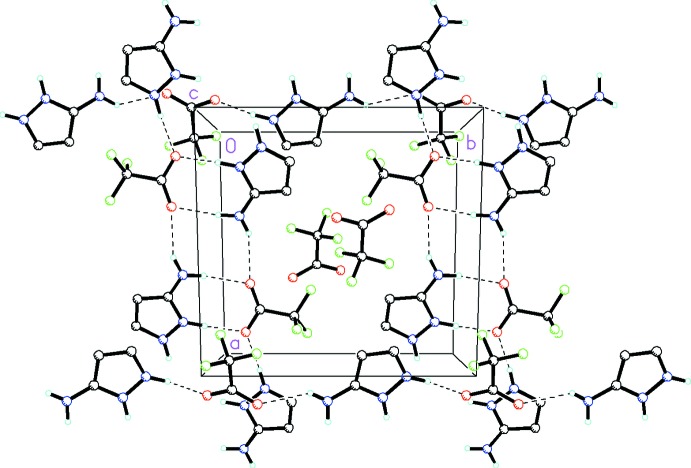
The crystal packing of the title compound, showing the hydrogen bonds (dashed lines) forming chains along [100] and [010]. H atoms not involved in hydrogen bonding and the minor component of disorder have been removed for clarity.

## supplementary crystallographic information

### 1. Comment

Pyrazoles are an important class of heterocyclic compounds and many pyrazole derivatives are reported to have a broad spectrum of biological properties, e.g. antibacterial and anti-inflammatory activities (Patel *et al.*, 2010), anticancer (Hall *et al.*, 2008), antimicrobial (Samshuddin *et al.*, 2010), anti-inflammatory, antidepressant, anticonvulsant and anti-HIV properties (Isloor *et al.*, 2009). The chemistry of aminopyrazoles has been extensively investigated in the past (Giuseppe *et al.*, 1991). The considerable biological and medicinal activities of pyrazoles (Vinogradov *et al.*, 1994) for which aminopyrazoles are preferred precursors, have stimulated our investigations.

The crystal structures of some related compounds, viz., 3-aminopyrazole-4-carboxylic acid (Dobson & Gerkin, 1998), 4-(3,5-dimethylpyrazol-1-yl)benzoic acid trifluoroacetate (Foces-Foces *et al.*, 1996), 2-amino-5-methylpyridinium trifluoroacetate (Thanigaimani *et al.*, 2012) and 2-amino-5-chloropyridinium trifluoroacetate (Hemamalini & Fun, 2010) have been reported. In view of the importance of the title compound this paper reports its crystal structure.

The asymmetric unit of the title compound consists of two crystallographically independent 3-aminopyrazolium cations (A and B) and two trifluoroacetate anions (A and B) (Fig. 1). Each 3-aminopyrazolium cation is planar, with a maximum deviation of 0.0006 (2) Å for atom N2A in cation A and 0.0005 (2) Å for atom N2B in cation B. In the cations, atoms N3A and N3B are protonated. The F atoms of both anions are disordered over two sets of positions, with occupancy ratios of 0.639 (12):0.361 (12). Bond lengths and are normal (Allen *et al.*, 1987).

In the crystal packing (Fig. 2), the A/B type 3-aminopyrazolium cations interact with the carboxylate groups of the A/B type trifluoroacetate anions through N—H···O hydrogen bonds, forming R_2_^2^(8), R_2_^4^(8), R_2_^4^(10), R_4_^4^(16) and R_4_^4^(18) (Bernstein *et al.*, 1995) ring motifs.

### 2. Experimental

A mixture of commercially available 3-aminopyrazole and trifluoroacetic acid (1:3 v/v) were stirred for 15 minutes at room temperature. X-ray quality crystals were formed on slow evaporation. (m.p.: 463-468 K).

### 3. Refinement

All H atoms were located in a difference Fourier map and refined independently with isotropic displacement parameters [N—H = 0.84 (3)–0.95 (3) Å and C—H = 0.89 (3)–0.96 (3)Å].

### Crystal data

**Table:**

C_3_H_6_N_3_^+^·C_2_F_3_O_2_^−^	*F*(000) = 800
*M**_r_* = 197.13	*D*_x_ = 1.681 Mg m^−^^3^
Monoclinic, *P*2_1_/*n*	Cu *K*α radiation, λ = 1.5418 Å
*a* = 10.9292 (8) Å	Cell parameters from 2544 reflections
*b* = 10.9332 (6) Å	θ = 3.4–72.4°
*c* = 13.7002 (13) Å	µ = 1.58 mm^−^^1^
β = 107.939 (9)°	*T* = 173 K
*V* = 1557.5 (2) Å^3^	Irregular, colourless
*Z* = 8	0.16 × 0.14 × 0.06 mm

### Data collection

**Table:**

Agilent Xcalibur (Eos, Gemini) diffractometer	3031 independent reflections
Radiation source: Enhance (Cu) X-ray Source	2343 reflections with *I* > 2σ(*I*)
Graphite monochromator	*R*_int_ = 0.030
Detector resolution: 16.0416 pixels mm^-1^	θ_max_ = 72.5°, θ_min_ = 4.6°
ω scans	*h* = −13→13
Absorption correction: multi-scan (*CrysAlis PRO* and *CrysAlis RED*; Agilent, 2012)	*k* = −13→9
*T*_min_ = 0.662, *T*_max_ = 1.000	*l* = −15→16
9227 measured reflections	

### Refinement

**Table:**

Refinement on *F*^2^	Primary atom site location: inferred from neighbouring sites
Least-squares matrix: full	Hydrogen site location: difference Fourier map
*R*[*F*^2^ > 2σ(*F*^2^)] = 0.048	All H-atom parameters refined
*wR*(*F*^2^) = 0.136	*w* = 1/[σ^2^(*F*_o_^2^) + (0.0697*P*)^2^ + 0.4559*P*] where *P* = (*F*_o_^2^ + 2*F*_c_^2^)/3
*S* = 1.05	(Δ/σ)_max_ < 0.001
3031 reflections	Δρ_max_ = 0.24 e Å^−^^3^
338 parameters	Δρ_min_ = −0.23 e Å^−^^3^
0 restraints	

### Special details

**Table:**

Geometry. All esds (except the esd in the dihedral angle between two l.s. planes) are estimated using the full covariance matrix. The cell esds are taken into account individually in the estimation of esds in distances, angles and torsion angles; correlations between esds in cell parameters are only used when they are defined by crystal symmetry. An approximate (isotropic) treatment of cell esds is used for estimating esds involving l.s. planes.

### Fractional atomic coordinates and isotropic or equivalent isotropic displacement parameters (Å^2^)

**Table:**

	*x*	*y*	*z*	*U*_iso_*/*U*_eq_	Occ. (<1)
C1A	0.56299 (19)	0.93721 (18)	0.25897 (17)	0.0384 (5)	
C2A	0.4240 (2)	0.7896 (2)	0.25222 (19)	0.0453 (5)	
H2A	0.351 (2)	0.741 (2)	0.2498 (18)	0.050 (7)*	
C3A	0.4396 (2)	0.9121 (2)	0.26406 (18)	0.0428 (5)	
H3A	0.379 (2)	0.968 (2)	0.2737 (18)	0.050 (7)*	
N1A	0.6272 (2)	1.04305 (18)	0.2644 (2)	0.0588 (6)	
H1AA	0.593 (3)	1.108 (3)	0.278 (2)	0.057 (8)*	
H1AB	0.713 (3)	1.041 (3)	0.272 (2)	0.064 (8)*	
N2A	0.61502 (17)	0.83064 (15)	0.24439 (15)	0.0396 (4)	
H2AA	0.697 (3)	0.815 (3)	0.240 (2)	0.062 (8)*	
N3A	0.52875 (17)	0.73971 (17)	0.23916 (16)	0.0440 (5)	
H3AA	0.549 (3)	0.657 (3)	0.236 (2)	0.067 (8)*	
C1B	0.28710 (18)	0.17323 (18)	0.48905 (16)	0.0351 (4)	
C2B	0.1618 (2)	0.3114 (2)	0.5268 (2)	0.0451 (5)	
H2B	0.122 (2)	0.382 (2)	0.5441 (19)	0.050 (7)*	
C3B	0.2771 (2)	0.2975 (2)	0.50783 (18)	0.0410 (5)	
H3B	0.336 (3)	0.354 (3)	0.508 (2)	0.061 (8)*	
N1B	0.37852 (18)	0.11030 (18)	0.46300 (18)	0.0472 (5)	
H1BA	0.450 (3)	0.147 (2)	0.476 (2)	0.053 (7)*	
H1BB	0.375 (3)	0.029 (3)	0.472 (2)	0.060 (8)*	
N2B	0.18060 (15)	0.11865 (16)	0.49623 (14)	0.0372 (4)	
H2BA	0.164 (2)	0.034 (3)	0.4985 (19)	0.058 (8)*	
N3B	0.10420 (17)	0.20371 (16)	0.52047 (16)	0.0431 (4)	
H3BA	0.024 (3)	0.184 (2)	0.5222 (19)	0.051 (7)*	
C4A	0.58112 (19)	0.40965 (18)	0.26078 (18)	0.0409 (5)	
C5A	0.4574 (2)	0.4142 (2)	0.29303 (18)	0.0422 (5)	
F1A1	0.4274 (7)	0.3086 (7)	0.3249 (5)	0.0619 (13)	0.639 (12)
F1A2	0.3945 (12)	0.3073 (14)	0.2818 (12)	0.075 (3)	0.361 (12)
F2A1	0.3589 (8)	0.4515 (8)	0.2173 (6)	0.0681 (17)	0.639 (12)
F2A2	0.3687 (14)	0.4932 (10)	0.2363 (13)	0.067 (3)	0.361 (12)
F3A1	0.4720 (7)	0.4955 (5)	0.3687 (6)	0.0651 (13)	0.639 (12)
F3A2	0.4800 (14)	0.4446 (15)	0.3885 (10)	0.089 (4)	0.361 (12)
O1A	0.63921 (15)	0.31128 (13)	0.27322 (15)	0.0526 (5)	
O2A	0.60816 (16)	0.50747 (14)	0.22685 (17)	0.0622 (5)	
C4B	0.23375 (19)	0.8115 (2)	0.48329 (18)	0.0413 (5)	
C5B	0.2075 (2)	0.6750 (2)	0.45833 (19)	0.0447 (5)	
F1B1	0.1329 (4)	0.6255 (3)	0.5045 (6)	0.077 (2)	0.639 (12)
F1B2	0.2028 (16)	0.6103 (8)	0.5367 (6)	0.101 (4)	0.361 (12)
F2B1	0.3162 (3)	0.6097 (3)	0.4866 (5)	0.0669 (13)	0.639 (12)
F2B2	0.2821 (9)	0.6190 (6)	0.4167 (12)	0.089 (4)	0.361 (12)
F3B1	0.1581 (7)	0.6629 (3)	0.3597 (3)	0.090 (2)	0.639 (12)
F3B2	0.0855 (7)	0.6537 (6)	0.3935 (8)	0.079 (3)	0.361 (12)
O1B	0.13834 (14)	0.86991 (13)	0.49109 (15)	0.0530 (5)	
O2B	0.34134 (14)	0.84860 (15)	0.48973 (15)	0.0538 (5)	

### Atomic displacement parameters (Å^2^)

**Table:**

	*U*^11^	*U*^22^	*U*^33^	*U*^12^	*U*^13^	*U*^23^
C1A	0.0374 (10)	0.0299 (10)	0.0508 (12)	0.0050 (8)	0.0177 (9)	0.0021 (8)
C2A	0.0330 (11)	0.0431 (13)	0.0629 (15)	−0.0022 (9)	0.0194 (10)	0.0043 (10)
C3A	0.0351 (10)	0.0382 (12)	0.0595 (14)	0.0072 (9)	0.0208 (10)	0.0022 (10)
N1A	0.0434 (11)	0.0275 (10)	0.113 (2)	0.0008 (8)	0.0358 (12)	−0.0044 (10)
N2A	0.0340 (9)	0.0284 (9)	0.0624 (12)	0.0006 (7)	0.0238 (8)	0.0023 (8)
N3A	0.0385 (9)	0.0281 (9)	0.0701 (13)	−0.0011 (7)	0.0239 (9)	0.0017 (8)
C1B	0.0265 (9)	0.0353 (11)	0.0453 (11)	−0.0029 (7)	0.0138 (8)	0.0041 (8)
C2B	0.0464 (12)	0.0299 (11)	0.0663 (15)	−0.0029 (9)	0.0280 (11)	−0.0050 (10)
C3B	0.0344 (10)	0.0341 (11)	0.0570 (13)	−0.0096 (8)	0.0178 (9)	−0.0010 (9)
N1B	0.0302 (9)	0.0350 (10)	0.0823 (15)	−0.0018 (8)	0.0259 (9)	0.0024 (9)
N2B	0.0288 (8)	0.0279 (9)	0.0588 (11)	−0.0013 (6)	0.0192 (7)	−0.0009 (7)
N3B	0.0345 (9)	0.0330 (9)	0.0709 (13)	−0.0024 (7)	0.0298 (9)	−0.0039 (8)
C4A	0.0354 (10)	0.0301 (11)	0.0639 (14)	−0.0021 (8)	0.0252 (10)	−0.0036 (9)
C5A	0.0363 (11)	0.0397 (12)	0.0549 (13)	−0.0014 (9)	0.0201 (10)	0.0010 (9)
F1A1	0.053 (3)	0.0486 (17)	0.099 (4)	−0.004 (2)	0.045 (3)	0.015 (3)
F1A2	0.046 (5)	0.054 (3)	0.135 (10)	−0.010 (4)	0.044 (5)	0.013 (7)
F2A1	0.0376 (17)	0.094 (5)	0.071 (2)	0.011 (3)	0.0149 (16)	0.019 (3)
F2A2	0.038 (4)	0.058 (5)	0.113 (8)	0.009 (4)	0.035 (5)	0.018 (4)
F3A1	0.061 (2)	0.067 (3)	0.079 (4)	−0.001 (2)	0.039 (2)	−0.027 (2)
F3A2	0.070 (4)	0.137 (11)	0.069 (5)	−0.006 (7)	0.034 (3)	−0.015 (7)
O1A	0.0452 (9)	0.0277 (8)	0.0968 (13)	0.0022 (6)	0.0394 (9)	0.0030 (7)
O2A	0.0526 (10)	0.0302 (8)	0.1222 (16)	0.0029 (7)	0.0538 (11)	0.0110 (9)
C4B	0.0309 (10)	0.0341 (11)	0.0634 (14)	0.0007 (8)	0.0211 (10)	0.0020 (9)
C5B	0.0379 (11)	0.0350 (12)	0.0636 (15)	0.0034 (9)	0.0193 (10)	−0.0015 (10)
F1B1	0.071 (2)	0.0309 (14)	0.151 (6)	−0.0066 (15)	0.068 (3)	0.005 (2)
F1B2	0.174 (11)	0.053 (4)	0.069 (4)	−0.026 (6)	0.025 (6)	0.011 (3)
F2B1	0.0527 (15)	0.0417 (14)	0.107 (3)	0.0162 (11)	0.0249 (19)	−0.0071 (17)
F2B2	0.072 (6)	0.059 (3)	0.163 (11)	−0.002 (3)	0.076 (7)	−0.038 (5)
F3B1	0.113 (5)	0.0658 (19)	0.069 (2)	−0.017 (2)	−0.003 (2)	−0.0142 (14)
F3B2	0.050 (3)	0.060 (3)	0.108 (6)	0.002 (2)	−0.003 (3)	−0.033 (3)
O1B	0.0343 (8)	0.0290 (8)	0.1059 (14)	−0.0020 (6)	0.0366 (8)	−0.0059 (8)
O2B	0.0310 (8)	0.0434 (9)	0.0937 (13)	−0.0013 (6)	0.0290 (8)	0.0035 (8)

### Geometric parameters (Å, º)

**Table:**

C1A—C3A	1.399 (3)	N2B—H2BA	0.95 (3)
C1A—N1A	1.344 (3)	N2B—N3B	1.358 (2)
C1A—N2A	1.338 (3)	N3B—H3BA	0.91 (3)
C2A—H2A	0.96 (3)	C4A—C5A	1.547 (3)
C2A—C3A	1.354 (3)	C4A—O1A	1.234 (2)
C2A—N3A	1.329 (3)	C4A—O2A	1.238 (3)
C3A—H3A	0.94 (3)	C5A—F1A1	1.312 (8)
N1A—H1AA	0.85 (3)	C5A—F1A2	1.341 (14)
N1A—H1AB	0.91 (3)	C5A—F2A1	1.309 (8)
N2A—H2AA	0.94 (3)	C5A—F2A2	1.351 (15)
N2A—N3A	1.357 (2)	C5A—F3A1	1.337 (7)
N3A—H3AA	0.93 (3)	C5A—F3A2	1.298 (14)
C1B—C3B	1.393 (3)	C4B—C5B	1.538 (3)
C1B—N1B	1.349 (3)	C4B—O1B	1.255 (2)
C1B—N2B	1.338 (2)	C4B—O2B	1.221 (2)
C2B—H2B	0.95 (3)	C5B—F1B1	1.295 (5)
C2B—C3B	1.372 (3)	C5B—F1B2	1.299 (8)
C2B—N3B	1.325 (3)	C5B—F2B1	1.337 (4)
C3B—H3B	0.89 (3)	C5B—F2B2	1.284 (6)
N1B—H1BA	0.84 (3)	C5B—F3B1	1.298 (4)
N1B—H1BB	0.90 (3)	C5B—F3B2	1.375 (6)
			
N1A—C1A—C3A	131.41 (19)	C2B—N3B—N2B	108.01 (17)
N2A—C1A—C3A	107.28 (18)	C2B—N3B—H3BA	130.5 (16)
N2A—C1A—N1A	121.30 (19)	N2B—N3B—H3BA	121.1 (16)
C3A—C2A—H2A	129.0 (15)	O1A—C4A—C5A	116.52 (18)
N3A—C2A—H2A	121.0 (15)	O1A—C4A—O2A	129.27 (19)
N3A—C2A—C3A	109.9 (2)	O2A—C4A—C5A	114.21 (17)
C1A—C3A—H3A	127.7 (15)	F1A1—C5A—C4A	113.4 (4)
C2A—C3A—C1A	106.02 (19)	F1A1—C5A—F3A1	108.0 (4)
C2A—C3A—H3A	126.3 (15)	F1A2—C5A—C4A	113.7 (7)
C1A—N1A—H1AA	118.4 (19)	F1A2—C5A—F2A2	103.9 (7)
C1A—N1A—H1AB	119.1 (18)	F2A1—C5A—C4A	111.2 (4)
H1AA—N1A—H1AB	120 (3)	F2A1—C5A—F1A1	108.0 (4)
C1A—N2A—H2AA	128.8 (18)	F2A1—C5A—F3A1	106.2 (4)
C1A—N2A—N3A	109.00 (17)	F2A2—C5A—C4A	113.1 (7)
N3A—N2A—H2AA	122.2 (18)	F3A1—C5A—C4A	109.7 (3)
C2A—N3A—N2A	107.79 (18)	F3A2—C5A—C4A	112.6 (6)
C2A—N3A—H3AA	129.0 (18)	F3A2—C5A—F1A2	105.7 (7)
N2A—N3A—H3AA	122.6 (18)	F3A2—C5A—F2A2	107.3 (8)
N1B—C1B—C3B	130.75 (19)	O1B—C4B—C5B	114.19 (18)
N2B—C1B—C3B	107.58 (17)	O2B—C4B—C5B	116.60 (19)
N2B—C1B—N1B	121.60 (19)	O2B—C4B—O1B	129.2 (2)
C3B—C2B—H2B	131.0 (15)	F1B1—C5B—C4B	113.5 (3)
N3B—C2B—H2B	119.5 (15)	F1B1—C5B—F2B1	105.8 (3)
N3B—C2B—C3B	109.57 (19)	F1B1—C5B—F3B1	110.1 (3)
C1B—C3B—H3B	125.5 (18)	F1B2—C5B—C4B	113.3 (4)
C2B—C3B—C1B	105.75 (18)	F1B2—C5B—F3B2	99.4 (5)
C2B—C3B—H3B	128.7 (18)	F2B1—C5B—C4B	111.4 (2)
C1B—N1B—H1BA	114.8 (18)	F2B2—C5B—C4B	117.5 (3)
C1B—N1B—H1BB	113.5 (17)	F2B2—C5B—F1B2	107.4 (6)
H1BA—N1B—H1BB	121 (2)	F2B2—C5B—F3B2	104.7 (5)
C1B—N2B—H2BA	128.4 (16)	F3B1—C5B—C4B	108.6 (2)
C1B—N2B—N3B	109.08 (17)	F3B1—C5B—F2B1	107.2 (3)
N3B—N2B—H2BA	121.5 (16)	F3B2—C5B—C4B	112.7 (3)
			
C1A—N2A—N3A—C2A	−1.0 (3)	O1A—C4A—C5A—F3A2	88.7 (8)
C3A—C1A—N2A—N3A	0.6 (3)	O2A—C4A—C5A—F1A1	177.4 (4)
C3A—C2A—N3A—N2A	1.1 (3)	O2A—C4A—C5A—F1A2	149.4 (7)
N1A—C1A—C3A—C2A	178.8 (3)	O2A—C4A—C5A—F2A1	55.5 (5)
N1A—C1A—N2A—N3A	−178.3 (2)	O2A—C4A—C5A—F2A2	31.3 (7)
N2A—C1A—C3A—C2A	0.1 (3)	O2A—C4A—C5A—F3A1	−61.7 (4)
N3A—C2A—C3A—C1A	−0.7 (3)	O2A—C4A—C5A—F3A2	−90.5 (8)
C1B—N2B—N3B—C2B	1.0 (3)	O1B—C4B—C5B—F1B1	−38.2 (4)
C3B—C1B—N2B—N3B	−0.9 (2)	O1B—C4B—C5B—F1B2	−76.1 (9)
C3B—C2B—N3B—N2B	−0.6 (3)	O1B—C4B—C5B—F2B1	−157.5 (3)
N1B—C1B—C3B—C2B	177.5 (2)	O1B—C4B—C5B—F2B2	157.7 (8)
N1B—C1B—N2B—N3B	−178.2 (2)	O1B—C4B—C5B—F3B1	84.6 (5)
N2B—C1B—C3B—C2B	0.5 (3)	O1B—C4B—C5B—F3B2	35.9 (7)
N3B—C2B—C3B—C1B	0.1 (3)	O2B—C4B—C5B—F1B1	143.9 (4)
O1A—C4A—C5A—F1A1	−3.4 (4)	O2B—C4B—C5B—F1B2	106.0 (9)
O1A—C4A—C5A—F1A2	−31.4 (7)	O2B—C4B—C5B—F2B1	24.5 (4)
O1A—C4A—C5A—F2A1	−125.3 (4)	O2B—C4B—C5B—F2B2	−20.3 (9)
O1A—C4A—C5A—F2A2	−149.5 (6)	O2B—C4B—C5B—F3B1	−93.3 (5)
O1A—C4A—C5A—F3A1	117.5 (4)	O2B—C4B—C5B—F3B2	−142.1 (6)

### Hydrogen-bond geometry (Å, º)

**Table:**

*D*—H···*A*	*D*—H	H···*A*	*D*···*A*	*D*—H···*A*
N1*A*—H1*AA*···O1*A*^i^	0.85 (3)	2.28 (3)	2.936 (3)	134 (2)
N1*A*—H1*AB*···O2*A*^ii^	0.91 (3)	1.99 (3)	2.884 (3)	169 (3)
N2*A*—H2*AA*···O1*A*^ii^	0.94 (3)	1.85 (3)	2.778 (2)	171 (3)
N3*A*—H3*AA*···O2*A*	0.93 (3)	1.78 (3)	2.705 (2)	172 (3)
N1*B*—H1*BA*···O2*B*^iii^	0.84 (3)	2.18 (3)	2.962 (2)	153 (2)
N1*B*—H1*BB*···O2*B*^iv^	0.90 (3)	2.03 (3)	2.929 (3)	173 (2)
N2*B*—H2*BA*···O1*B*^iv^	0.95 (3)	1.81 (3)	2.756 (2)	174 (2)
N3*B*—H3*BA*···O1*B*^v^	0.91 (3)	1.82 (3)	2.728 (2)	171 (2)

Symmetry codes: (i) *x*, *y*+1, *z*; (ii) −*x*+3/2, *y*+1/2, −*z*+1/2; (iii) −*x*+1, −*y*+1, −*z*+1; (iv) *x*, *y*−1, *z*; (v) −*x*, −*y*+1, −*z*+1.

## References

[bb1] Agilent (2012). *CrysAlis PRO* and *CrysAlis RED* Agilent Technologies, Yarnton, England.

[bb2] Allen, F. H., Kennard, O., Watson, D. G., Brammer, L., Orpen, A. G. & Taylor, R. (1987). *J. Chem. Soc. Perkin Trans. 2*, pp. S1–19.

[bb3] Bernstein, J., Davis, R. E., Shimoni, L. & Chang, N.-L. (1995). *Angew. Chem. Int. Ed. Engl.* **34**, 1555–1573.

[bb4] Dobson, A. J. & Gerkin, R. E. (1998). *Acta Cryst.* C**54**, 253–256. 10.1107/s0108270197013115 9540201

[bb5] Dolomanov, O. V., Bourhis, L. J., Gildea, R. J., Howard, J. A. K. & Puschmann, H. (2009). *J. Appl. Cryst.* **42**, 339–341.

[bb6] Foces-Foces, C., Cativiela, C., Zurbano, M. M., Sobrados, I., Jagerovic, N. & Elguero, J. (1996). *J. Chem. Crystallogr.* **26**, 579–584.

[bb7] Giuseppe, D., Salvatore, P. & Demetrio, R. (1991). *Trends Heterocycl. Chem.* **2**, 97.

[bb8] Hall, A., Billinton, A., Brown, S. H., Clayton, N. M., Chowdhury, A., Gerald, M. P., Goldsmith, G. P., Hayhow, T. G., Hurst, D. N., Kilford, I. R., Naylor, A. & Passingham, B. (2008). *Bioorg. Med. Chem. Lett.* **18**, 3392–3399. 10.1016/j.bmcl.2008.04.018 18462938

[bb9] Hemamalini, M. & Fun, H.-K. (2010). *Acta Cryst.* E**66**, o783–o784. 10.1107/S1600536810008196 PMC2983973 21580623

[bb10] Isloor, A. M., Kalluraya, B. & Shetty, P. (2009). *Eur. J. Med. Chem.* **44**, 3784–3787. 10.1016/j.ejmech.2009.04.038 19464087

[bb11] Palatinus, L. & Chapuis, G. (2007). *J. Appl. Cryst.* **40**, 786–790.

[bb12] Patel, C. K., Rami, C. S., Panigrahi, B. & Patel, C. N. (2010). *J. Chem. Pharm. Res.* **2**, 73–78.

[bb13] Samshuddin, S., Narayana, B., Yathirajan, H. S., Safwan, A. P. & Tiekink, E. R. T. (2010). *Acta Cryst.* E**66**, o1279–o1280. 10.1107/S1600536810015795 PMC2979444 21579379

[bb14] Sheldrick, G. M. (2008). *Acta Cryst.* A**64**, 112–122. 10.1107/S0108767307043930 18156677

[bb15] Thanigaimani, K., Farhadikoutenaei, A., Khalib, N. C., Arshad, S. & Razak, I. A. (2012). *Acta Cryst.* E**68**, o3319–o3320. 10.1107/S1600536812045291 PMC3588927 23476163

[bb16] Vinogradov, V. M., Dalinger, I. L. & Shevelev, S. A. (1994). *Khim. Farm. Zh.* **28**, 37–46.

